# Highlighting the achievements and impact of women in data science

**DOI:** 10.1016/j.patter.2025.101263

**Published:** 2025-06-13

**Authors:** Lauren Higa, Youping Deng

**Affiliations:** 1 Department of Quantitative Health Sciences, John A. Burns School of Medicine, University of Hawaii at Manoa, Honolulu, HI 96813, USA; 2 Molecular Biosciences and Bioengineering Program, College of Tropical Agriculture and Human Resources, University of Hawaii at Manoa, Honolulu, HI 96822, USA; 3 Genomics and Bioinformatics Shared Resource, University of Hawaii Cancer Center, Honolulu, HI 96813, USA; 4 Pacific Center for Genome Research, University of Hawaii Cancer Center, Honolulu, HI 96813, USA

## Abstract

Women have been instrumental in shaping data science from its earliest days. This opinion highlights both the achievements and the ongoing challenges faced by women in the field, emphasizing that a wide range of perspectives and backgrounds among data scientists is essential to drive innovation and improve research quality.

## Main text

### Introduction

Women have had a profound impact on the field of data science. From Ada Lovelace, who laid the groundwork for modern computing, to Fei-Fei Li, who has made advancements in artificial intelligence, women have consistently been at the forefront of innovation. In our lab, we have been fortunate to work alongside talented women whose contributions to data science have inspired both new and experienced researchers alike. Collectively, these achievements highlight the importance of fostering an environment where women are not only welcomed but equipped to thrive, ensuring that their unique perspectives and expertise continue to shape the future of data science.

### Historical contributions of women in data science

The history of data science is deeply intertwined with the contributions of women, many of whom have been overlooked or undervalued. Women have been foundational to the field’s development since its earliest days as mathematicians, statisticians, and coders who shaped how we analyze and interpret data. For example, Ada Lovelace is widely recognized as the first computer programmer. In the 1840s, Lovelace translated and annotated an article about the analytical engine, which was a proposed mechanical digital computer designed by Charles Babbage.^1^ Her detailed notes on the article were the first to suggest the potential of machines to go far beyond calculation and predicted that machines would go on to, someday, manipulate symbols, logic, and data. This led Lovelace to develop a nearly complete program for calculating Bernoulli numbers with the proposed analytical engine. She even envisioned that such machines would one day be capable of creating music or art, parallel to modern-day artificial intelligence.

Over a decade later, Florence Nightingale led a major healthcare reform for British soldiers during the Crimean War.^2^ After realizing that preventable diseases were a major cause of soldier mortality, she began collecting and analyzing battlefield hospital data. To persuade the British military to improve hospital conditions, Nightingale and her team used innovative statistical diagrams to convey these findings. Her efforts were among the first in history to exemplify how data visualization could be used to push for policy change and save lives.

In the mid-20^th^ century, mathematicians Katherine Johnson, Dorothy Vaughan, and Mary Jackson were instrumental in NASA’s space missions and the United States’ efforts during the Space Race. They had crucial roles in calculating orbital mechanics, including the flight path of the first American astronaut in orbit and the trajectory for the Apollo 11 moon landing.^3^ As Black women in the mid-20^th^ century, they were subject to discrimination and segregation in the workplace. However, despite these major barriers, they made invaluable contributions to some of NASA’s greatest accomplishments using mathematical modeling and early computer programming.

Around the same time, Grace Hopper was a rear admiral in the US Navy and a trailblazer of modern computer programming. Hopper is largely credited for the development of the FLOW-MATIC programming language and COBOL, a high-level English-like programming language that is still in use today.^4^ Hopper’s work helped bridge the gap between computer code and human-readable languages, which was an essential step in making computers more accessible for data science work.

More recently, Fei-Fei Li has played a pivotal role in the advancement of artificial intelligence, particularly in the field of deep learning. She is best known for creating ImageNet, a large-scale visual dataset that transformed computer vision. ImageNet allowed deep neural networks to be trained on an unprecedented scale, and its use in the 2012 ImageNet Challenge demonstrated that these models could significantly outperform traditional computer vision methods.^5^ This breakthrough has contributed to major progress in areas such as autonomous vehicles, facial recognition, and medical image analysis.

From Ada Lovelace’s early vision of computer programming to Fei-Fei Li’s impact on modern artificial intelligence, women have continually pushed the boundaries of data science. Recognizing the contributions of these women is essential not only to honoring their legacy but also to building a more inclusive and innovative future in data science.

### Current gender disparities in data science

Despite these achievements, women remain significantly underrepresented in data science. According to a 2024 report by Anaconda, women account for only 23% of data science professionals globally, While this disparity cannot be attributed to a single factor, there are well-documented gender barriers that hinder women’s progress in scientific fields. Multiple studies have shown that gender bias influences hiring practices, salaries, and professional evaluations, with women often receiving lower pay and fewer opportunities than equally qualified male counterparts.^6^^,^^7^ Research has also found that, although historically underrepresented scholars contribute innovative ideas, their work is adopted at a lower rate.^8^ Furthermore, women are less likely to receive proper recognition for their contributions, particularly in the form of authorship in academic publications, perpetuating a perceived gap in productivity.^9^ These challenges highlight the urgent need for targeted initiatives and systemic changes to create a more supportive and equitable environment for women in data science.

### The significance of women in data science

The value of women’s perspectives in data science cannot be overstated. Data science is fundamentally about understanding and interpreting the world through data, and when teams lack a wide range of experiences and perspectives, the resulting insights are inevitably limited. Women bring unique viewpoints that can greatly enhance data science approaches. One of the most pressing issues in data science is the risk of biased algorithms. When data science teams consist of people with similar backgrounds and experiences, it can result in biased algorithms that reflect or perpetuate societal biases. Research has shown that prediction errors correlate within demographic groups; however, combining predictions across multiple demographic groups—including gender—tends to improve overall algorithmic performance.^10^ This suggests that the different perspectives in diverse teams lead to more accurate outcomes.

Besides reducing algorithmic bias, diversity drives innovation. Research has consistently shown that teams with a mix of backgrounds produce more creative and effective solutions.^8^ In data science, where innovation is the key to addressing complex problems, diversity is a major advantage. Women’s perspectives may challenge conventional thinking, leading to breakthroughs that less-inclusive teams might miss.

Lastly, representation matters. As demonstrated by Florence Nightingale, data science has the power to shape public policy, healthcare, and even societal norms. However, women have often been excluded from important discussions, research, and policy decisions that directly affect their lives. Without their voices, data-driven solutions risk overlooking the needs of women. Hence, a proper representation of women in data science teams ensures that these solutions consider a wider range of community concerns.

Ultimately, the inclusion of women in data science is necessary to advance the field. Their contributions lead to more accurate, innovative, and impactful outcomes that are better suited to tackle the complex challenges of our world. Therefore, the field of data science must cultivate an inclusive and supportive environment where women are equipped to thrive at every stage of their careers.

### Developing an equitable environment for women in data science

Increasing the representation of women in data science requires sustained action and systemic change. Institutions and organizations must provide tangible support through targeted mentorship programs, dedicated funding, and training to address unconscious biases.^11^^,^^12^ By establishing clear and effective policies that prevent discrimination and promote work-life balance, such as flexible work hours and parental leave, we can create a safe and supportive workplace for women. Furthermore, institutions should actively promote the visibility and recognition of women’s contributions by ensuring fair authorship opportunities, competitive salaries, transparent promotion pathways, and unbiased consideration for awards. These concrete measures are essential not only for fostering an environment where women can fully contribute to the field of data science but also for dismantling systemic barriers that have historically disadvantaged women.

### Conclusion

Women bring unique perspectives and insights that enrich data-driven research and lead to more innovative and impactful outcomes. Their contributions help prevent biases in algorithm development, drive innovation through new avenues of thinking, and ensure that the outcomes of data science better reflect the needs of the community. By implementing meaningful measures such as mentorship programs, proper recognition, and enforcement of antidiscrimination policies, institutions can create an environment where more women are empowered to prosper. Ultimately, fostering a culture of inclusivity in data science refines research quality, improves decision-making, and strengthens the impact of data science on society.

## Acknowledgments

This work was partially supported by the National Institutes of Health grants P20GM103466, U54MD007601, U54GM138062, U54HG013243, T32DK137523, and UE5HG013826.

## Declaration of interests

Y.D. serves as an advisory board member for *Patterns*.
